# Errata

**DOI:** 10.36660/abc.20230356

**Published:** 2023-07-19

Edição de Setembro de 2022, vol. 119(3), págs. 393-399

No Artigo Original “O Valor Preditivo do Escore CHA2DS2-VASc no Escore Syntax Residual em Pacientes com Infarto do Miocárdio com Supradesnivelamento do Segmento ST”, com número de DOI: https://doi.org/10.36660/abc.20210670, publicado no periódico Arquivos Brasileiros de Cardiologia, Arq Bras Cardiol. 2022; 119(3):393-399, na página 1, alterar o nome do autor correspondente para: Ali Kemal Kalkan e o email do autor correspondente para: drakkalkan@gmail.com

Edição de Fevereiro de 2021, vol. 116(2), págs. 219-226

No Artigo Original “Resultados Clínicos e Hemodinâmicos de Longo Prazo após o Transplante de Coração em Pacientes Pré-Tratados com Sildenafil”, com número de DOI: https://doi.org/10.36660/abc.20190047, publicado no periódico Arquivos Brasileiros de Cardiologia, Arq Bras Cardiol. 2021; 116(2):219-226, na página 225, corrigir em Fontes de Financiamento para: O presente estudo foi parcialmente financiado pela Agência de Fomento de Pesquisa FCT POCI-01-0145-FEDER-032414.

