# Supplementary material for: Errata
Source: Arq Bras Cardiol. 2023 Jul 19;120(7):e20230356. [Article in Portuguese] doi: 10.36660/abc.20230356 (PMC10374261; doi:10.36660/abc.20230356)
Supplement: Translation

# Translation: Errata

September 2022 issue, vol. 119(3), pages 393-399

In the Original Article “The Predictive Value of CHA2DS2-VASc Score on Residual Syntax Score in Patients With ST Segment Elevation Myocardial Infarction”, with DOI: https://doi.org/10.36660/abc.20210670, published in the journal Arquivos Brasileiros de Cardiologia, Arq Bras Cardiol. 2022; 119(3):393-399, on page 1, change the corresponding author name to: Ali Kemal Kalkan and the corresponding author email to: drakkalkan@gmail.com.

February 2021 issue, vol. 116(2), pages 219-226

In the Original Article “Long-Term Clinical and Hemodynamic Outcomes after Heart Transplantation in Patients Pre-Treated with Sildenafil”, with DOI: https://doi.org/10.36660/abc.20190047, published in the journal Arquivos Brasileiros de Cardiologia, Arq Bras Cardiol. 2021; 116(2):219-226, on page 225, correct under Sources of Funding to: This study was partially funded by the FCT Research Development Agency POCI-01-0145-FEDER-032414.
